# Multiple diffusion metrics in differentiating solid glioma from brain inflammation

**DOI:** 10.3389/fnins.2023.1320296

**Published:** 2024-01-30

**Authors:** Kai Zhao, Ankang Gao, Eryuan Gao, Jinbo Qi, Ting Chen, Guohua Zhao, Gaoyang Zhao, Peipei Wang, Weijian Wang, Jie Bai, Yong Zhang, Huiting Zhang, Guang Yang, Xiaoyue Ma, Jingliang Cheng

**Affiliations:** ^1^Department of Magnetic Resonance Imaging, The First Affiliated Hospital of Zhengzhou University, Zhengzhou, China; ^2^MR Research Collaboration, Siemens Healthineers Ltd., Wuhan, China; ^3^Shanghai Key Laboratory of Magnetic Resonance, East China Normal University, Shanghai, China

**Keywords:** magnetic resonance imaging, non-Gaussian, diffusion-weighted imaging, glioma, brain inflammation

## Abstract

**Background and purpose:**

The differential diagnosis between solid glioma and brain inflammation is necessary but sometimes difficult. We assessed the effectiveness of multiple diffusion metrics of diffusion-weighted imaging (DWI) in differentiating solid glioma from brain inflammation and compared the diagnostic performance of different DWI models.

**Materials and methods:**

Participants diagnosed with either glioma or brain inflammation with a solid lesion on MRI were enrolled in this prospective study from May 2016 to April 2023. Diffusion-weighted imaging was performed using a spin-echo echo-planar imaging sequence with five b values (500, 1,000, 1,500, 2000, and 2,500 s/mm^2^) in 30 directions for each b value, and one b value of 0 was included. The mean values of multiple diffusion metrics based on diffusion tensor imaging (DTI), diffusion kurtosis imaging (DKI), mean apparent propagator (MAP), and neurite orientation dispersion and density imaging (NODDI) in the abnormal signal area were calculated. Comparisons between glioma and inflammation were performed. The area under the curve (AUC) of the receiver operating characteristic curve (ROC) of diffusion metrics were calculated.

**Results:**

57 patients (39 patients with glioma and 18 patients with inflammation) were finally included. MAP model, with its metric non-Gaussianity (NG), shows the greatest diagnostic performance (AUC = 0.879) for differentiation of inflammation and glioma with atypical MRI manifestation. The AUC of DKI model, with its metric mean kurtosis (MK) are comparable to NG (AUC = 0.855), followed by NODDI model with intracellular volume fraction (ICVF) (AUC = 0.825). The lowest value was obtained in DTI with mean diffusivity (MD) (AUC = 0.758).

**Conclusion:**

Multiple diffusion metrics can be used in differentiation of inflammation and solid glioma. Non-Gaussianity (NG) from mean apparent propagator (MAP) model shows the greatest diagnostic performance for differentiation of inflammation and glioma.

## 1 Introduction

Glioma is the most common primary brain tumor and requires timely surgical treatment for a better prognosis (Lapointe et al., 2018). Brain inflammation, on the other hand, is a common benign lesion with associated neurologic dysfunction and non-operative therapy as the main treatment (Hodler et al., 2020). Early identification of glioma from inflammation is essential. However, these two types of diseases sometimes overlap in clinical symptoms, signs, and laboratory tests (Han et al., 2021).

Currently, the preoperative diagnosis of glioma relies on magnetic resonance imaging (MRI) examination (Zoccarato et al., 2019). Some cystic or necrotic brain inflammation may exhibit ring-shaped enhancement, making it prone to misdiagnosis as glioblastoma, consequently leading to erroneous treatment decisions (Sabel et al., 2001; Nadal Desbarats et al., 2003). Central necrosis, hemorrhage, and ring-shaped enhancement are considered typical malignant features of high-grade gliomas in advanced stages. These characteristics are associated with rapid tumor cell growth, inadequate blood and oxygen supply to the tumor core, damage to the blood–brain barrier and immature angiogenesis. Several studies (Hiremath et al., 2017; Bo et al., 2021) have advanced imaging analysis methods to distinguish gliomas exhibiting typical malignant features from conditions such as brain abscesses and tumefactive demyelination. On the other hand, gliomas with atypical MRI presentations are prone to misdiagnosis as brain inflammation, resulting in treatment delays and further tumor progression, thereby worsening prognosis (Talathi et al., 2015; Lu et al., 2019), resulting in treatment delays and further progression, thereby worsening prognosis. Some research (Wu et al., 2021; Piao et al., 2022) have defined atypical MRI manifestation of glioma as the absence of an obvious mass effect or enhancement. Gliomas with such atypical manifestations are deemed challenging to differentiate from brain inflammation using conventional MRI, making them a focal point for research. We recognize the importance of choosing cases with comparable imaging presentations for studies on imaging methods that aim to distinguish between gliomas and brain inflammation. This strategy aligns with the pragmatic considerations of clinical practice (Omuro et al., 2006). However, the definition of atypical MRI manifestations in gliomas remains ambiguous and lacks standardized criteria.

Building upon the studies and case reports mentioned above, we advocate for the incorporation of a straightforward and widely applicable set of selection criteria in investigations of novel imaging techniques. This involves including glioma cases based on the identification of either cystic or solid lesions as primary criteria. Additionally, the selection of cases of brain inflammation with comparable imaging presentations is emphasized to establish a homogeneous control group. Specifically, distinguishing cystic/necrotic gliomas from cerebral abscesses, cysticercosis or tumefactive demyelination, and differentiating solid gliomas from brain inflammation with similar imaging presentations.

Accurate diagnosis of solid lesions is paramount, as these manifest in the early stages of the disease. Timely intervention can curtail lesion progression, preserve cerebral function, and enhance overall prognosis. Recent research (Wu et al., 2021; Piao et al., 2022) found that the deep learning and radiomics analysis based on conventional MRI performed well in distinguishing glioma and brain inflammation, but the features extracted by those methods are limited in characterizing the pathophysiological and microstructural differences between two type of lesions due to their complex numerical nature (Abdel Razek et al., 2021). Therefore, the use of advanced MRI techniques to access patients with suspected glioma is in need.

Diffusion-weighted imaging (DWI) is increasingly used because of its ability to quantitatively assess the microstructure of lesion. Advanced diffusion models describe the displacement of the water molecules more accurately, which can illustrate the microstructural information of the tissue better. Several non-Gaussian diffusion models have been used to evaluate glioma, and they performed well in predicting glioma genotyping (Gao et al., 2022) and distinguishing glioblastoma from solitary brain metastasis (Qi et al., 2022; Wang et al., 2022). In this study, we evaluated the performance of 4 diffusion models in differentiating glioma with atypical MRI manifestation from brain inflammation, including diffusion tensor imaging (DTI), diffusion kurtosis imaging (DKI), mean apparent propagator (MAP), and neurite orientation dispersion and density imaging (NODDI) models.

## 2 Materials and methods

The study was approved by scientific research and clinical trial ethics committee of the first affiliated hospital of Zhengzhou university, and informed consent was waived (Approval Number: 2019-KY-231).

### 2.1 Patients

This retrospective study involved the collection of imaging data from 62 patients diagnosed with either glioma or inflammation from May 2016 to April 2023. The inclusion criteria were: (1) glioma histopathologically confirmed cerebral gliomas based on the World Health Organization (WHO) 2021 classification criteria or brain inflammation confirmed through pathological biopsy or cerebrospinal fluid analysis; (2) MRI shows a solid lesion without hemorrhagic, ring-shaped enhancement, or patchy heterogeneous signals of necrosis.

The exclusion criteria were: (1) patients who had undergone surgery, anti-tumor therapy, steroids or anti-infective treatment before the MRI examination; (2) MRI images with severe susceptibility artifacts or motion artifacts; (3) lesions located under the tentorium of cerebellum; (4) incomplete imaging data.

### 2.2 MRI protocol

All patients underwent MRI scans on a 3 T MR scanner (MAGNETOM Prisma, Siemens Healthineers, Erlangen, Germany) with a 64 channel of head–neck coil. The acquisition sequence and parameters were as follows: (1). T1WI: repetition time (TR), 250.0 ms; excitation time (TE), 2.46 ms; number of slices, 20; slice thickness, 5.0 mm; field of view (FOV), 220 × 220 mm^2^; acquisition matrix, 314 × 314; (2). T2WI: TR, 4,090.0 ms; TE, 99.0 ms; number of slices, 20; slice thickness, 5.0 mm; FOV, 220 × 220 mm^2^; acquisition matrix, 733 × 733; (3). T2 dark-fluid: TR, 8,000.0 ms; TE, 81.0 ms; number of slices, 20; slice thickness, 5.0 mm; FOV, 220 × 220 mm^2^; acquisition matrix, 314 × 314; (4). DWI: spin-echo echo-planar imaging sequence, TR 2,500 ms, TE 71 ms, number of slices, 60; slice thickness, 2.2 mm; FOV, 220 × 220 mm^2^, five non-zero b values (500, 1,000, 1,500, 2000, and 2,500 s/mm^2^) with 30 directions for every b value, and one zero b value (b = 0 s/mm^2^).

### 2.3 Diffusion-weighted imaging processing

Eddy current and motion correction were conducted on diffusion-weighted data using the Diffusion Kit Eddy tool^1^ (Xie et al., 2016). The DWI images were processed by NeuDiLab (Diffusion Imaging in Python)^2^ to obtain b = 0 s/mm^2^ (b0) image and the metric maps including the DKI-based mean kurtosis (MK), the DTI-based mean diffusivity (MD) and fractional anisotropy (FA), the MAP-based mean squared displacement (MSD), *q*-space inverse variance (QIV), non-Gaussianity (NG) and return-to-origin probability (RTOP), the NODDI-based intracellular volume fraction (ICVF) and orientation dispersion index (ODI).

### 2.4 Image processing and analysis

**Figure 1:**
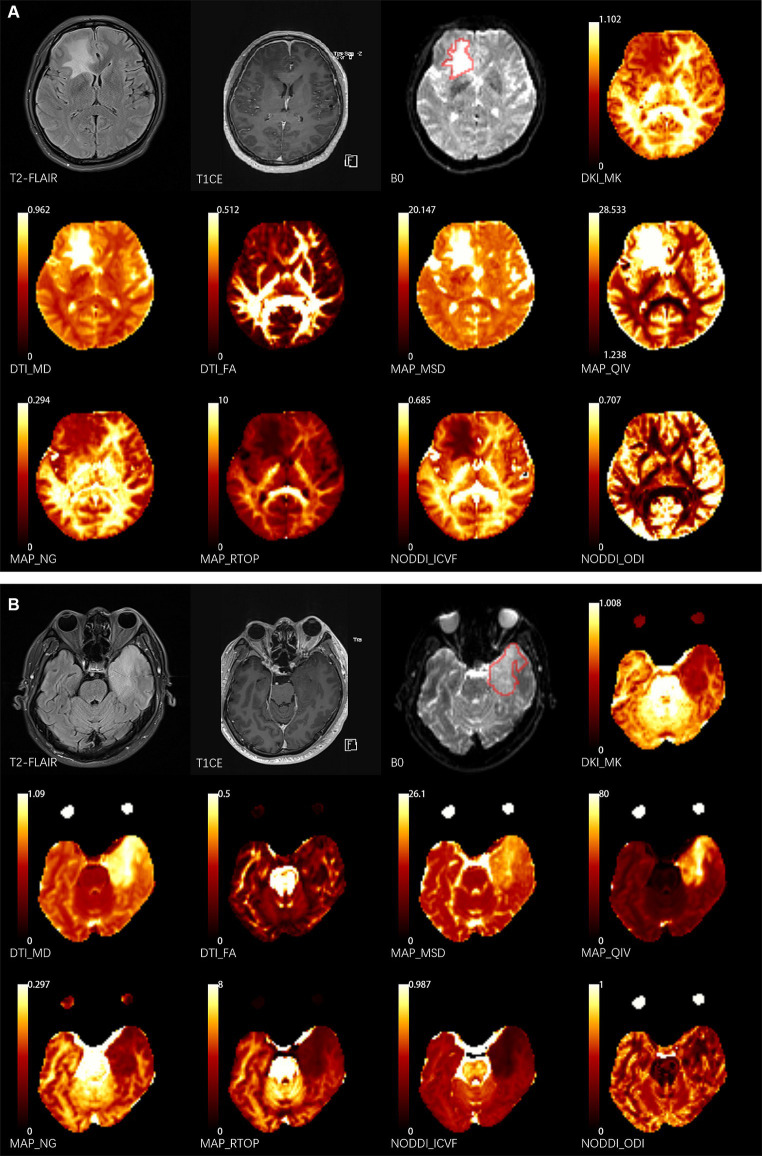
Two representative cases. The delineation of the volume of interest (VOI) is shown by the red lines on the b0 images. **(A)** 43-year-old female patient presents with NMDA-IgG positivity autoimmune encephalitis. **(B)** A 52-year-old male patient with left temporal glioblastoma (World Health Organization grade 4). T1CE, T1 weighted contrast enhancement; DKI, diffusion-kurtosis imaging; MK, mean kurtosis; DTI, diffusion-tensor imaging; MD, mean diffusivity; FA, fractional anisotropy; MAP, mean apparent propagator; MSD, Mean squared diffusion; NG, mean non-Gaussianity; RTOP, return-to-origin probability; QIV, *q*-space inverse variance; NODDI, neurite orientation dispersion and density imaging; ICVF, intracellular volume fraction; ODI, orientation dispersion index.

The volumes of interest (VOIs) of lesions were delineated using ITK-SNAP software^3^ by two neuroradiologists (K.Z. and X.M., 3 and 11 years of experience, respectively) who were blind to the diagnostic information. The VOIs of lesions were defined as abnormal hyperintense signals on the b0 image (Figure 1) and cerebrospinal fluid signals were avoided. Since the b0 images were part of the DWI sequence, it was simple to align VOIs with other metric maps (Huang et al., 2021). The mean value of each metric map was calculated by FAE^4^ (Song et al., 2020).

### 2.5 Statistical analysis

Statistical analyses were all performed by software environment R (v4.2.0).^5^ The chi-square test was used to compare the sex distribution of the patients between the two groups. The normality of the data and homogeneity of the variance were evaluated using the Shapiro–Wilk and Levene’s tests, respectively. The differences of the metrics and mean age between glioma and inflammation were compared using independent *t*-test or Mann–Whitney *U* test depending on the results of test for normality and homoscedasticity. All data were expressed as the mean ± standard deviation (SD) or median (25th percentile, 75th percentile) depending on the test method. Cohen’s d effect sizes were calculated to demonstrate the strength of difference between parameters in inflammation group and glioma group. A value of Cohen’s d greater than 0.8 was considered as a large effect size (Ma et al., 2020). The receiver operating characteristic (ROC) curve was drawn and the area under the curve (AUC), sensitivity, specificity and accuracy were calculated to evaluate the diagnostic performance of each metric. The optimal cut-off values were selected based on the best Youden Index. Delong test was used to compare the differential diagnostic performance. Statistical significance was set at *p* < 0.05.

## 3 Results

### 3.1 Patients characteristics

**Figure 2:**
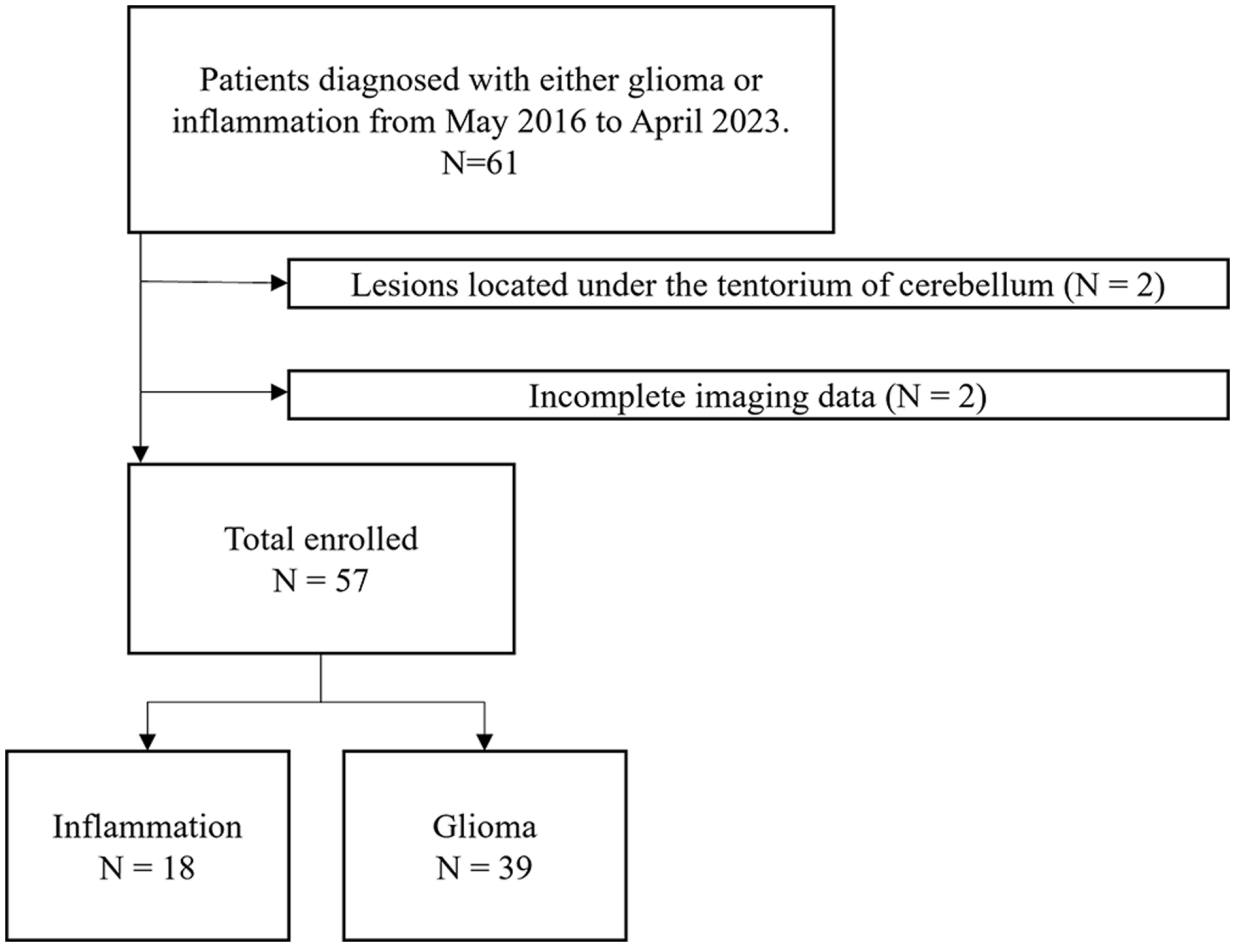
Participant selection flowchart.

The demographic characteristics and the timepoint of imaging in relation to symptom onset of included patients were summarized in Table 1. Overall, 57 participants (34 men, 23 women, mean age, 46 years; age range, 17–73 years) were included in this study (Figure 2). 24 participants were diagnosed with WHO grade 2 glioma (10 astrocytoma, 12 oligodendroglioma, 2 Not Otherwise Specified (NOS)), 11 participants were diagnosed with WHO grade 3 glioma (4 astrocytoma, 5 oligodendroglioma, 2 NOS), 4 participants were diagnosed with WHO grade 4 glioma (4 glioblastoma). 18 participants were diagnosed with brain inflammation. The average age of patients in the inflammation group is significantly higher than that of those in the glioma group (*p* < 0.05). There were no significant differences in gender or onset between two groups (*p* > 0.05).

**Table 1:** Patient characteristics.

	Total	Inflammation	Glioma	*p*
Age	46 ± 12	52 ± 10	43 ± 13	0.006
Sex				0.112
Male	34	8	26	
Female	23	10	13	
Onset				0.511
Acute (< 2 weeks)	21	7	14	
Subacute (2 weeks–3 months)	18	7	11	
Chronic (> 3 months)	18	4	14	

### 3.2 Histogram analyses of DWI parameters

**Figure 3:**
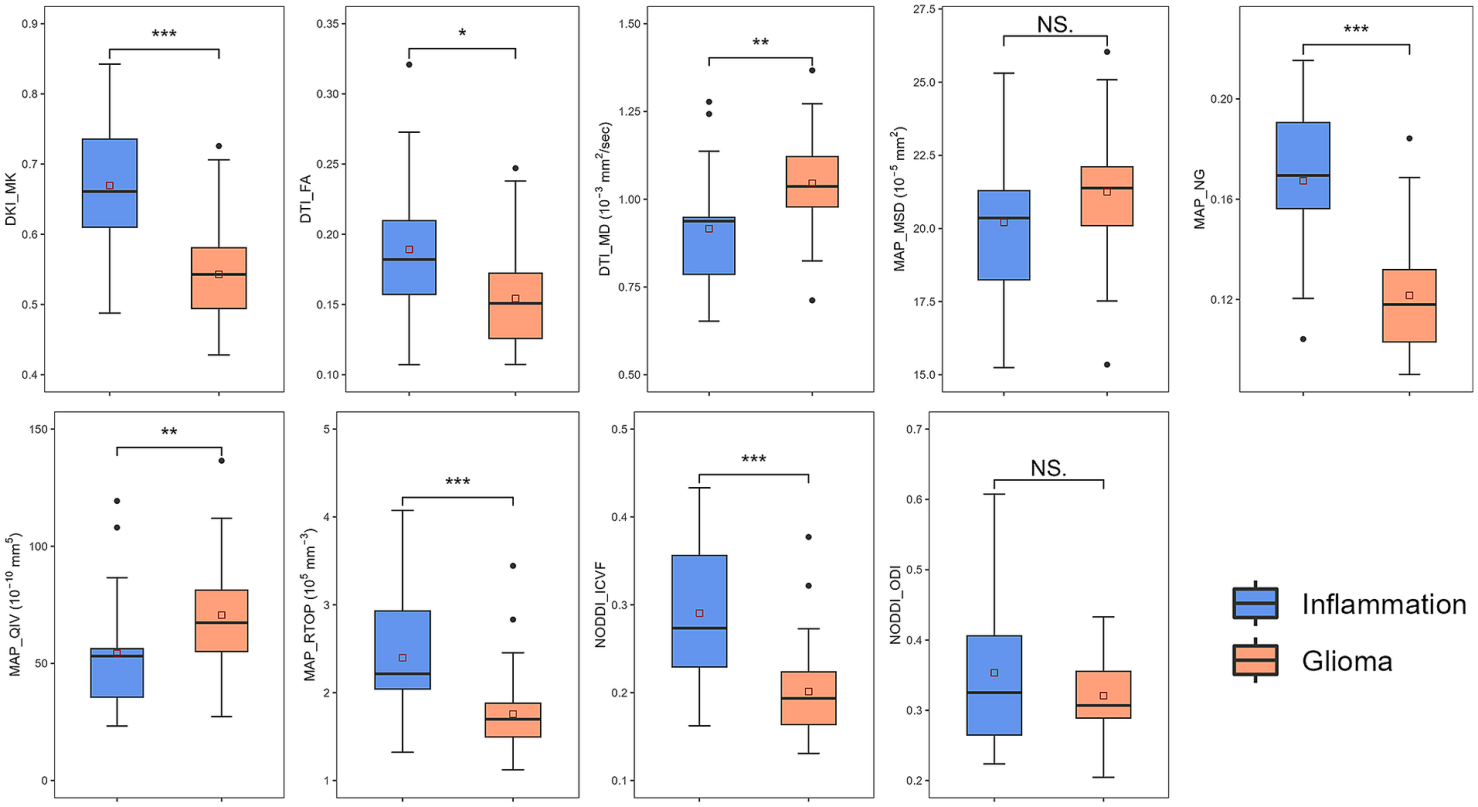
Boxplots of diffusion metrics. * represented *p* = 0.01 ~ 0.05, ** represented *p* = 0.001 ~ 0.01, *** represented *p* ≤ 0.001. DKI, diffusion-kurtosis imaging; MK, mean kurtosis; DTI, diffusion-tensor imaging; MD, mean diffusivity; FA, fractional anisotropy; MAP, mean apparent propagator; MSD, Mean squared diffusion; NG, mean non-Gaussianity; RTOP, return-to-origin probability; QIV, q-space inverse variance; NODDI, neurite orientation dispersion and density imaging; ICVF, intracellular volume fraction; ODI, orientation dispersion index.

Various metrics differed significantly between glioma group and inflammation group (Table 2). MK, NG, FA, RTOP, and ICVF were significantly lower in the glioma compared with those in the inflammation (*p* < 0.05); MD, MSD, QIV were significantly higher in the glioma compared with those in the inflammation (*p* < 0.05). There were no significant differences in ODI between two groups (*p* > 0.05). Corresponding boxplots of metrics were shown in Figure 3.

**Table 2:** Mean values of diffusion metrics of inflammation and glioma.

	Inflammation	Glioma	*t*/z	*p*	Cohen’s d
MK	0.669 ± 0.098	0.543 ± 0.066	5.769*	< 0.001	−1.644
FA	0.181 (0.153,0.208)	0.151 (0.126,0.172)	477	0.030	−0.693
MD	0.916 ± 0.169	1.045 ± 0.127	−3.218*	0.002	0.917
MSD	20.208 ± 2.623	21.251 ± 2.142	−1.592*	0.117	0.454
NG	0.169 (0.156,0.191)	0.118 (0.103,0.132)	617	< 0.001	−1.836
QIV	53.126 (35.598,56.28)	67.366 (55.093,81.27)	181	0.003	0.71
RTOP	2.215 (2.041,2.931)	1.699 (1.497,1.881)	558	< 0.001	−1.195
ICVF	0.273 (0.229,0.356)	0.194 (0.164,0.224)	579	< 0.001	−1.422
ODI	0.325 (0.265,0.406)	0.307 (0.289,0.355)	369	0.766	−0.432

^*^In line with normal distribution, independent *t*-test was adopted.

### 3.3 Performance of diagnosis

**Figure 4:**
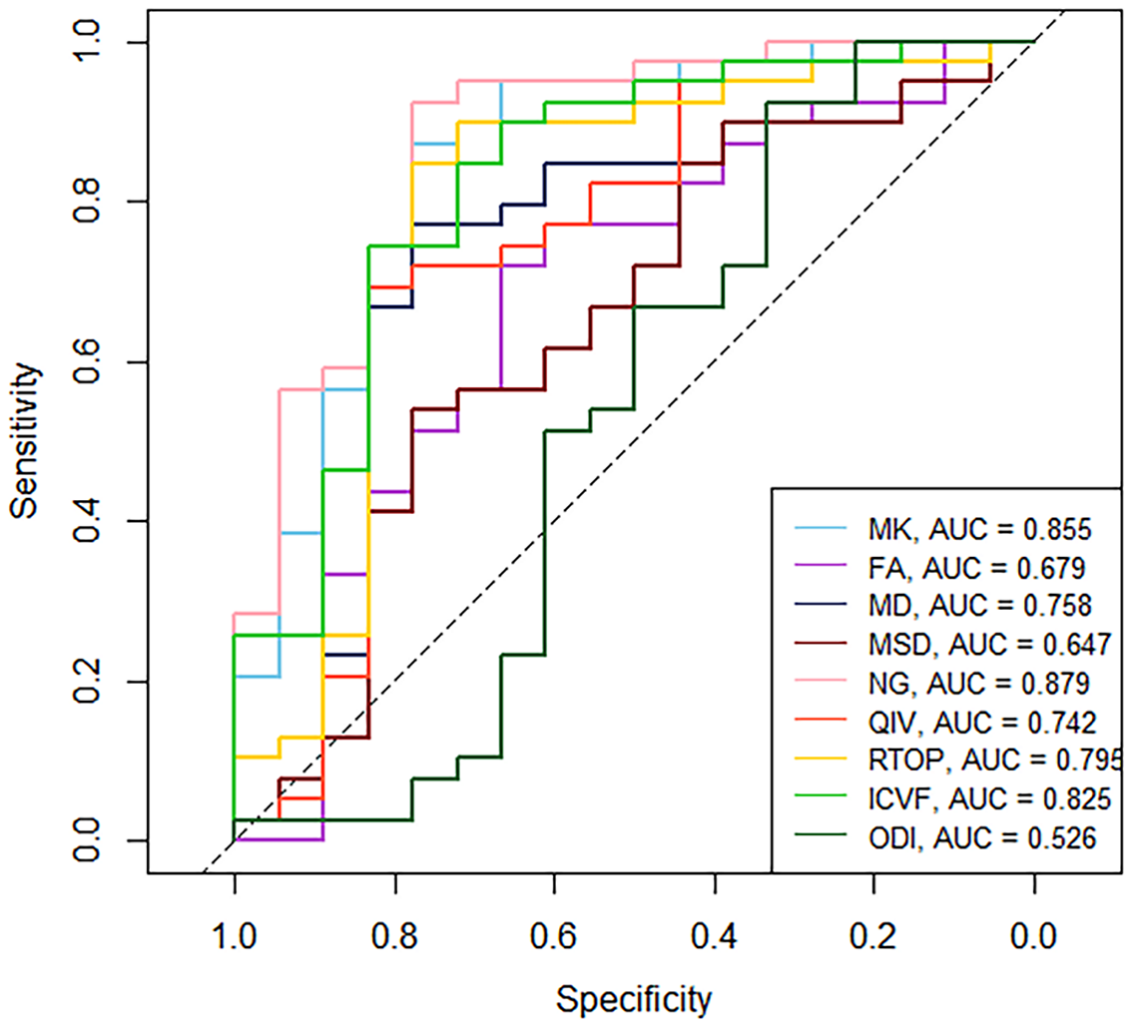
ROC curves of the diffusion metrics for distinguishing glioma from brain inflammation. MK, mean kurtosis; MD, mean diffusivity; FA, fractional anisotropy; MSD, Mean squared diffusion; NG, mean non-Gaussianity; RTOP, return-to-origin probability; QIV, q-space inverse variance; ICVF, intracellular volume fraction; ODI, orientation dispersion index.

Table 3 and Figure 4 present the results of the ROC curve analyses of diffusion metrics. The NG derived from MAP model had highest AUC value. Based on the Delong test (Table 4), a comparison of the area under the curve (AUC) for the most valuable diagnostic parameters among different models revealed NG demonstrates the highest AUC, significantly surpassing both ICVF and MD. There is no significant difference observed in AUC between NG and MK. MK follows as the second-highest, with a significant increase in AUC compared to MD. There is no significant difference in AUC between MK and ICVF. ICVF exhibits a significantly higher AUC compared to MD.

**Table 3:** ROC curve analysis of diffusion metrics for differentiation of inflammation and glioma.

	Cut-off	AUC (95%CI)	*p*	Sensitivity	Specificity	Accuracy
MK	0.600	0.855 (0.737,0.972)	< 0.001	0.778	0.872	0.842
FA	0.171	0.679 (0.516,0.843)	0.016	0.667	0.718	0.702
MD	0.962	0.758 (0.599,0.917)	< 0.001	0.778	0.769	0.772
MSD	21.3	0.647 (0.482,0.812)	0.041	0.778	0.538	0.614
NG	0.150	0.879 (0.776,0.982)	< 0.001	0.778	0.923	0.877
QIV	61.1	0.742 (0.582,0.902)	0.002	0.833	0.692	0.737
RTOP	2.01	0.795 (0.645,0.945)	< 0.001	0.778	0.846	0.825
ICVF	0.221	0.825 (0.698,0.951)	< 0.001	0.833	0.718	0.754
ODI	0.399	0.526 (0.331,0.72)	0.398	0.333	0.923	0.737

**Table 4:** Delong test for diffusion metrics with largest AUC in each model for differentiation of inflammation and glioma.

	*z*	*p*
MK vs. MD	2.927	0.003
MK vs. NG	−1.762	0.078
MK vs. ICVF	1.908	0.056
MD vs. NG	−2.995	0.003
MD vs. ICVF	−2.547	0.011
NG vs. ICVF	2.315	0.021

## 4 Discussion

Our study aimed to assess the discriminative potential of multiple diffusion metrics of diffusion-weighted imaging (DWI) in distinguishing solid glioma from inflammation. Various diffusion models, including diffusion-tensor imaging (DTI), diffusion-kurtosis imaging (DKI), neurite orientation dispersion and density imaging (NODDI), mean apparent propagator (MAP) were utilized. Our results demonstrated that the non-Gaussianity (NG) from MAP model may hold the greatest potential as a diffusion metric for differentiation of inflammation and glioma with the highest AUC (0.879) as well as the largest effect size (Cohen’s d = −1.644).

NG quantifies diffusion heterogeneity by assessing the divergence between the spin displacement probability density function (PDF) and its Gaussian approximation. Similar to NG, mean kurtosis (MK) is a measure of the deviation of water molecule movement from a Gaussian distribution within a tissue. In biological tissues, the diffusion behavior of water molecules is often influenced by various complex factors such as cell size and membrane permeability, resulting in non-Gaussian diffusion patterns. Both higher NG and MK value indicates a greater deviation from a Gaussian distribution, suggesting a more complex and heterogeneous microstructure of the tissue (Ozarslan et al., 2013). The similar physiological significance between NG and MK may explain the comparable diagnostic performance of the two (AUC of 0.879 and 0.855, Delong test *p* = 0.078). We initially hypothesized NG and MK to be larger in glioma for more diffusion barrier (Raab et al., 2010), as histopathological studies have revealed that due to loss of contact inhibition, gliomas exhibit higher degrees of cellularity and cytological atypia compared to reactive gliosis in brain inflammation (Hewer et al., 2020). However, the results were contrary to such assumption. In glioma, both the smaller NG and MK, as well as the larger MD and MSD, have indicated a smaller diffusion barrier than inflammation. One possible explanation is that the majority of the glioma cases with atypical MRI manifestation involved low-grade glioma (LGG). Tumor cell proliferation in LGG is characterized by larger cell volume, relatively smaller density, and reduced extracellular space due to extrusion between cells (Raab et al., 2010). Consequently, the barrier restricting the diffusion of water molecules including phospholipids and macromolecular proteins becomes less (Goryawala et al., 2018). Conversely, during the course of inflammation, the reparative response of brain tissue to injury may lead to an enhancement of its structural integrity. Zhuo et al. (2012) found that reactive gliosis has been shown to be a prominent feature during recovery from brain inflammation and this process can gradually increase the value of MK, which helps to support our view. Additionally, there is a discernable difference in the cellular morphology of benign and malignant gliosis. Research (Rivera-Zengotita and Yachnis, 2012) utilizing immunohistochemistry targeting glial fibrillary acidic protein (GFAP) have demonstrated that reactive astrocytes are found in an evenly spaced pattern with multiple thin, elongated radiating glial processes that extend into the stroma. In contrast, astrocytoma cells exhibit shorter and thicker processes (Shao et al., 2016). This disparity in astrocytic morphology may result in a higher cell membrane surface area within the voxel of benign glial hyperplasia, leading to the formation of more diffusion barriers that hinder gaussian diffusion of water molecules.

Furthermore, another explanation for the less diffusion restriction in gliomas is the more severe damage inflicted upon brain tissue by gliomas compared to inflammation. ICVF in NODDI model has been confirmed by histological studies (Jespersen et al., 2010) to exhibit a correlation with myelin staining. Our study found a lower ICVF value in glioma, which may be reflective of reduced intracellular diffusivity caused by more severe neuron injury or axonal loss (Chong et al., 2021). Also, the extracellular matrix produced by glioma may be another factor that reduces the density of white matter fibers and axons (Zamecnik, 2005). In DKI model, MK value reflects the complexity and structural integrity of brain tissue (Das et al., 2017). Previous studies on the application of DKI to low-grade gliomas (Goryawala et al., 2018) and inflammation (Liu et al., 2022) have demonstrated lower radial kurtosis (RK) values in lesions in comparison to healthy controls or contralateral normal-appearing white matter, which related to the destructive impact exerted by tumor cells or inflammation on brain tissue. In our research, the values of MK in glioma were lower, suggesting more severe structural damage in glioma than inflammatory lesions. In MAP model, NG has been identified as an indicator of the organizational complexity within tissues (Ozarslan et al., 2013). Meanwhile, RTOP has been shown to decrease in response to damage of neural fibers (Jiang et al., 2021b). Besides, in a recent study by Gao et al. (2022), it was discovered that values of NG and RTOP were significantly smaller in more invasive isocitrate dehydrogenase (IDH) wild-type gliomas compared to those with IDH mutant. Given the stronger invasiveness of IDH wild-type gliomas, these MAP metrics may potentially serve as parameters to characterize differences in lesion invasiveness. The invasion behavior and infiltration of glioma cells is a crucial factor affecting the diffusion metrics.

Fractional anisotropy (FA) is widely utilized to assess the coherence of white matter fiber bundles, our investigation found that FA in glioma was lower, which may represent more severe damage to white matter fiber bundles. A study (Hiremath et al., 2017) utilizing DTI to differentiate demyelination and glioma revealed no significant differences between the solid components and peritumoral regions of the two lesion groups (*p* = 0.341 and 0.052, respectively). These findings contradict our results, which could be attributed to the small sample size employed (*n* = 35). However, the AUC and the effect size of FA in our research were relatively small (AUC = 0.679, Cohen’s d = 0.693), indicating a lack of practical value (Ma et al., 2020). It is possible that the gaussian diffusion model-based limitations of FA in elucidating the intricate microstructural features of tissues may have had a bearing on this outcome (Chong et al., 2021). FA is influenced by both white matter fiber reduction and fiber crossing, which are difficult to differentiate using the DTI model, particularly in areas affected by edema (Jiang et al., 2021a). NODDI model is based on the three-compartment theory of non-Gaussian diffusion of water molecules, and it decomposes the physiological significance of FA into ICVF and orientation dispersion index (ODI) (Slattery et al., 2017). Histologically, ODI was found to be more correlated with orientation dispersion than FA, reflecting the dispersion of nerve walking, which could be used to characterize fiber crossing (Schilling et al., 2018). Results in our research showed no significant differences in Orientation Dispersion Index (ODI) between the two groups (*p* = 0.766), suggesting that fiber crossings and distortions occur in both inflammation and glioma, which limited the role of FA in characterizing white matter integrity.

In summary, non-Gaussian diffusion models, including MAP and DKI, have greater potential than NODDI and DTI for characterize the differences of microstructure, the extent of brain tissue damage and invasiveness between inflammation and glioma, thus facilitating their differential diagnosis. However, these advantages are based on technical principles and indirect results rather than direct pathological validation, highlighting the need for further research.

This study has several limitations that should be considered. Firstly, the sample size of cases with inflammation is relatively small, which may result in biased or inaccurate results. Secondly, the imbalanced proportion of different types of cases included in the study could further exacerbate this issue. Moreover, the VOIs were manually delineated in this study. This approach lacks objectivity and may introduce errors or inconsistencies in the data analysis. Alternative methods for identifying and measuring the VOIs, such as automated segmentation algorithms, might help mitigate this limitation in future studies.

## 5 Conclusion

Multiple diffusion metrics is a promising approach for distinguish solid glioma from inflammation. Non-Gaussianity (NG) from mean apparent propagator (MAP) model shows the greatest potential for differentiation of inflammation and glioma.

## Data availability statement

The raw data supporting the conclusions of this article will be made available by the authors, without undue reservation.

## Ethics statement

The studies involving humans were approved by the First Affiliated Hospital of Zhengzhou University. The studies were conducted in accordance with the local legislation and institutional requirements. Written informed consent for participation was not required for this study in accordance with the national legislation and the institutional requirements.

## Author contributions

KZ: Writing – original draft, Writing – review & editing. AG: Conceptualization, Funding acquisition, Writing – review & editing. EG: Data curation. TC: Data curation. GuZ: Software. GaZ: Data curation. PW: Software. WW: Funding acquisition. JB: Data curation. YZ: Writing – review & editing. HZ: Software. GY: Software. XM: Writing – review & editing, Funding acquisition. JC: Writing – review & editing.

## Footnotes

^1^https://diffusionkit.Readthedocs.io

^2^http://nipy.org/dipy

^3^http://www.itksnap.org/pmwiki/pmwiki.php

^4^https://github.com/salan668/FAE

^5^http://www.r-project.org
